# Ultrastructure and development of *Nosema podocotyloidis* n. sp. (Microsporidia), a hyperparasite of *Podocotyloides magnatestis* (Trematoda), a parasite of *Parapristipoma octolineatum* (Teleostei)

**DOI:** 10.1051/parasite/2014044

**Published:** 2014-09-02

**Authors:** Bhen Sikina Toguebaye, Yann Quilichini, Papa Mbagnick Diagne, Bernard Marchand

**Affiliations:** 1 Université Cheikh Anta Diop de Dakar, Laboratoire de Parasitologie, Faculté des Sciences et Techniques BP 5055 Dakar République du Sénégal; 2 CNRS – Université de Corse, UMR SPE 6134, Service d’Étude et de Recherche en Microscopie Électronique, Campus Grimaldi BP 52 20250 Corte Corse France; 3 Université Cheikh Anta Diop de Dakar, Laboratoire de Biologie Évolutive, d’Écologie et de Gestion des Écosystèmes, Faculté des Sciences et Techniques BP 5055 Dakar République du Sénégal

**Keywords:** *Nosema podocotyloidis*, Microsporidia, Hyperparasite, Digenea, *Podocotyloides magnatestis*, *Parapristipoma octolineatum*

## Abstract

*Nosema podocotyloidis* n. sp. (Microsporidia, Nosematidae) is described from *Podocotyloide*s *magnatestis* (Trematoda: Opecoelidae), a parasite of the fish *Parapristipoma octolineatum* (Teleostei) in the Atlantic Ocean. Electron microscopy reveals that all the stages of the cycle (merogony and sporogony) are diplokaryotic and in direct contact with the cytoplasm of host cells. There is no sporophorous vesicle (pansporoblast). The earliest stages observed are meronts, which have a simple plasmic membrane. Their cytoplasm is granular, rich in ribosomes and contains some sacculi of endoplasmic reticulum. They divide by binary fission into diplokaryotic sporonts. The sporonts have a thick electron-dense wall. Their diplokaryon is slightly less electron-dense than the cytoplasm. The cytoplasm of more advanced sporonts has numerous electron-lucent vesicles. Sporonts with two diplokarya divide by binary fission into diplokaryotic sporoblasts. The older sporoblasts are irregular or elongate and the polar filament is in formation. Their cytoplasm is denser, with ribosomes and lamellae of granular endoplasmic reticulum. The sporoblasts evolve into spores. The mature spores are broadly oval and measure 3.6 (3.1–4.0) × 2.58 (1.8–3.3) μm. Their wall is 100–300 nm thick. The polar tube is isofilar with 11–16 coils, 130–155 nm in diameter and arranged in many layers in the centre of the spore. The polaroplast is divided into two regions: an outer electron-dense cup with granular content and lacking lamellae and an internal region, less electron-dense, composed of irregularly arranged sacs. The posterior vacuole, with an amorphous electron-dense content, is present. The new species is compared with other species of *Nosema* from trematodes.

## Abstract (fr)

*Nosema podocotyloidis* n. sp. (Microsporidia, Nosematidae) est décrit de *Podocotyloide*s *magnatestis* (Trematoda : Opecoelidae), parasite du poisson *Parapristipoma octolineatum* (Teleostei) pêché dans l’Océan Atlantique. La microscopie électronique montre que tous les stades de développement (mérogonie et sporogonie) sont diplocaryotiques et en contact direct avec le cytoplasme des cellules hôtes. Il n’y a pas de pansporoblaste. Les plus jeunes stades observés sont des mérontes possédant une membrane plasmique simple. Leur cytoplasme est granulaire, riche en ribosomes et contient quelques saccules de réticulum endoplasmique. Ils donnent, par division binaire, des sporontes diplocaryotiques. Les sporontes sont recouverts d’une paroi épaisse dense aux électrons. Leur diplocaryon est légèrement moins dense que le cytoplasme qui, chez les sporontes âgés, présente de nombreuses vésicules claires aux électrons. Les sporontes avec deux diplocaryons donnent, par division binaire, des sporoblastes avec un diplocaryon. Les sporoblastes âgés sont allongés ou de forme irrégulière et présentent le tube polaire en formation. Leur cytoplasme est plus dense aux électrons et riche en ribosomes libres et en réticulum endoplasmique granulaire. Les sporoblastes évoluent en spores. Les spores matures sont grossièrement ovales et mesurent 3.6 (3.1-4.0) × 2.58 (1.8-3.3) μm. Leur paroi a une épaisseur de 100-300 nm. Le tube polaire est isofilaire, mesure 130-155 nm de diamètre et décrit 11-16 tours de spire disposés en plusieurs couches concentriques au niveau du centre de la spore. Le polaroplaste est divisé en deux régions : une externe, opaque aux électrons avec un contenu granulaire sans lamelles, et une région interne, moins opaque aux électrons, composée de saccules arrangés de façon irrégulière. La vacuole postérieure, avec un contenu amorphe opaque aux électrons est présente. La nouvelle espèce est comparée aux autres espèces de *Nosema* parasites de Trématodes.

## Introduction

*Podocotyloide*s *magnatestis* (Trematoda: Opecoelidae) is a parasite in the gut of the teleostean fish *Parapristipoma octolineatum* (Valenciennes, 1833) off the coast of Senegal. While studying this parasite, we found that some specimens were hyperparasitised by a microsporidia.

The microsporidia are unicellular eukaryotes and intracellular parasites. Their hosts include protists, invertebrates and all five classes of vertebrates [9, 22]. They are the earliest diverging clade of sequenced fungi [10]. Hyperparasitism in microsporidia is a known phenomenon. Microsporidia were found in gregarines [20, 22], myxosporidia [13], cestodes [19], dicyemids [12], copepods [18] and trematodes [7]. The occurrence of microsporidian parasites in trematodes is a relatively rare phenomenon and its detection is difficult. Some authors, in particular Sprague [21], Hussey [14], Canning [2], Canning et al. [3], Canning and Olson [7], Azevedo and Canning [1] and Levron et al. [16, 17], have reported this hyperparasitism. The well-described species of microsporidia known as natural hyperparasites of trematodes belong to the genus *Nosema* Naegeli, 1857 and the genus *Unikaryon* Canning, Lai and Lie, 1974 [1, 8].

The genus *Nosema* is identified by the following characters: all the stages of the cycle are diplokaryotic and in direct contact with the cytoplasm of host cells, merogony by binary division and ends with diplokaryotic sporonts, sporogony disposroblastic, spores diplokaryotic and generally ovoidal [9, 22].

The genus *Unikaryon* was established for the type species *Unikaryon piriformis*, a hyperparasite of *Echninoparyphium dunni* and *Echinostoma audyi*, both parasites of the snail *Lymnaea rubiginosa* [3, 4, 9]. The essential characters of this species are: nuclei are isolated at all stages of development, the sporogony is disposroblastic and the development occurs in contact with the host cell cytoplasm [5, 9, 23].

In addition to these two genera, there are some microsporidian species recorded in trematodes and classified in the genus *Pleistophora* Gurley, 1893 and in the collective group *Microsporidium* Balbiani, 1984. Unfortunately, these species have been very inadequately described and certain generic determinations are questionable. These species, listed by Sprague [22], are *Pleistophora* sp. Lie, Basch and Umathevy, 1966, *Microsporidium distomi* (Lutz and Splendore, 1908), *Microsporidium danilewskyi* (Pfeiffer, 1895) and *Micrsoporidium ghigii* (Guyénot and Naville, 1924).

For the genus *Pleistophora*, essential characters proposed by Canning and Nicholas [6] based on the ultrastructure of the type species *P. typicalis* from the marine fish *Myoxocephalus scorpius* are:

- nuclei are unpaired in all stages of the development;
- merogony stages are bounded by a dense and amorphous wall which detaches from the plasma membrane at the beginning of the sporogony forming the sporophorous vesicle wall;
- division of meronts takes place by plasmotomy;
- sporogony is polysporous by successive divisions of the sporogonial plasmodium until the formation of uninucleate sporoblasts which develop into spores;
- there are numerous spores in the sporophorous vesicle.

The collective group *Microsporidium* Balbiani, 1984 has been created by Sprague [22]. It regroups the species whose generic positions are uncertain.

In this paper, we describe a new species, assign it to the genus *Nosema* Naegeli, 1857, and compare it with the species of *Nosema* from trematodes previously studied using the same techniques.

## Materials and methods

Specimens of *Podocotyloides magnatestis* Aleshkina and Gaevskaya, 1985 (Trematoda, Digenea) were collected live from the intestine of naturally infested *Parapristipoma octolineatum* (Pisces, Teleostei, Actinopterygii), caught off Dakar (Atlantic Ocean).

The worms were removed from their hosts, fixed in cold (4 °C) 2.5% glutaraldehyde in 0.1M sodium cacodylate buffer at pH 7.2, rinsed in 0.1M sodium cacodylate buffer at pH 7.2, postfixed in cold (4 °C) 1% osmium tetroxyde in the same buffer for 1 h, dehydrated in ethanol and propylene oxide, embedded in Spurr and polymerised at 60 °C for 24 h.

Ultrathin sections (60–90 nm) were cut on an ultramicrotome (Power Tome PC, RMC Boeckeler). The sections were placed on grids and stained with uranyl acetate and lead citrate. Sections were examined on a Hitachi H-7650 transmission electron microscope, operating at an accelerating voltage of 80 kV, in the “Service d’Étude et de Recherche en Microscopie Électronique” of the University of Corsica (Corte, France).

## *Nosema podocotyloidis* n. sp.

urn:lsid:zoobank.org:act:B2161001-F580-4C29-9684-CC6E4CDB99E3

Type host: *Podocotyloides magnatestis* (Trematoda: Opecoelidae), a parasite of the teleost *Parapristipoma octolineatum.*

Type locality: Atlantic Ocean near Dakar (Senegal).

Type material: Hapantotype on grids No. 82689 deposited in the “Parasites et Écosystèmes Méditerranéens” Laboratory, University of Corsica (France).

Site of infection: Parenchyma.

Development: All stages of merogony and sporogony are diplokaryotic and in close contact with the host cell cytoplasm. Sporogony is diplosporoblastic.

Spores: Spores, in fixed preparations, broadly oval, 3.6 (3.1–4.0) × 2.58 (1.8–3.3) μm (*n* = 25). Spore wall 100–300 nm thick. Polar tube isofilar, 155 nm wide, arranged in 11–16 coils with multilayer structure. Polaroplast with an outer electron-dense cup with granular contents and lacking lamellae and an internal region, less electron-dense, composed of irregularly arranged sacs. Posterior vacuole with amorphous electron-dense content.

Etymology: After the host name.

### Description (Figs. 1–4)

**Figure 1.**
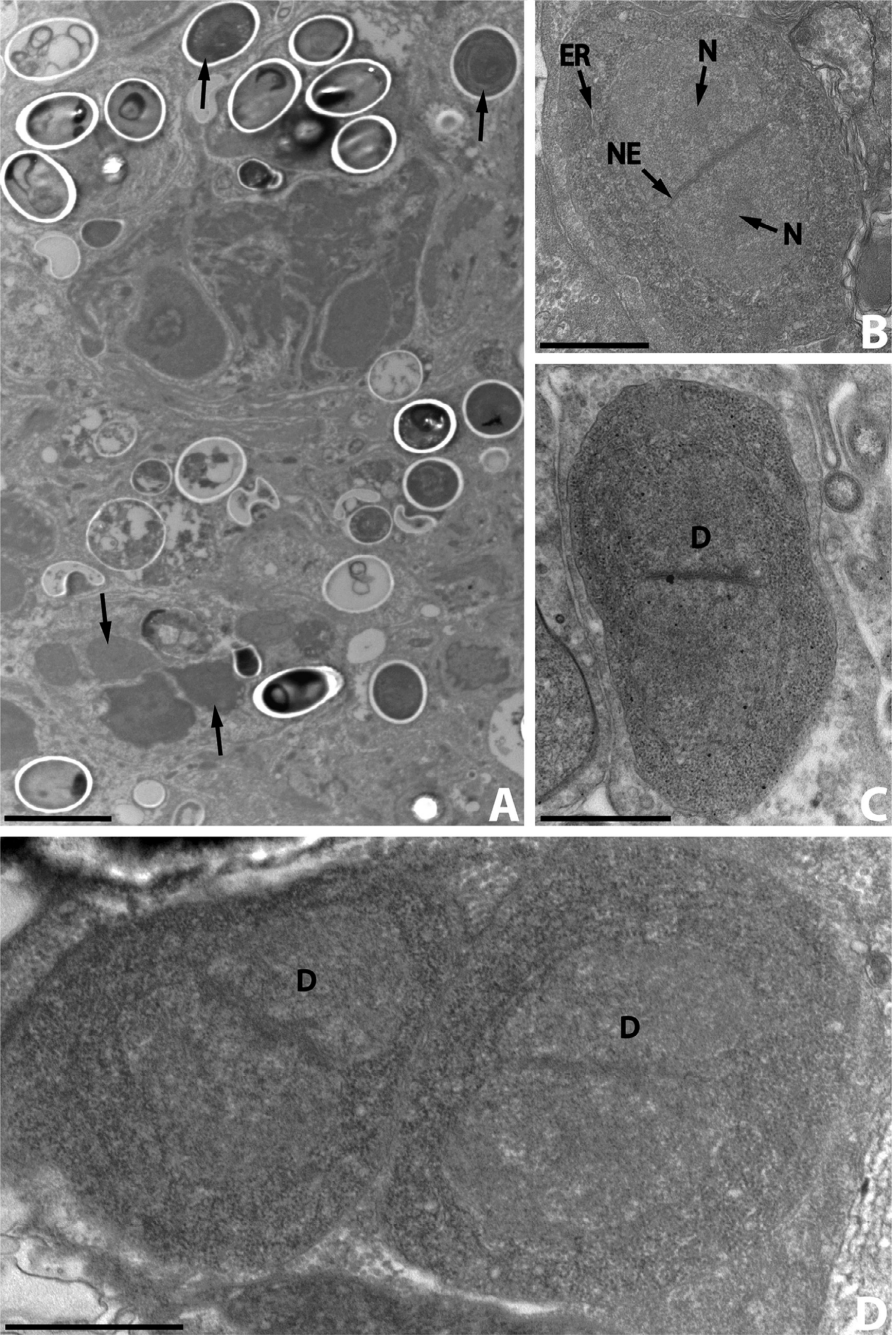
A. Ultrastructural aspects of the different developmental stages (arrows) of *Nosema podocotyloidis* n. sp. from *Podocotyloides magnatestis*. B. Ovoid meront. C. Elongate meront. D. Binary fission of meront with two diplokarya. D: diplokaryon; ER: endoplasmic reticulum; N: nucleolus; NE: nuclear envelope. Scale Bars: A, 5 μm; B, C and D, 1 μm.

All stages of merogony and sporogony observed were arranged diffusely in the host cell cytoplasm, without a sporophorous vesicle (Fig. 1A).

### Meronts and merogony

The merogonic stages were diplokaryotic cells with a thin plasmic membrane (Fig. 1B–D). They were in direct contact with the host cell cytoplasm. They were never seen in vacuoles. The cytoplasm was uniform, granular and contained a great number of free ribosomes. There were only traces of an endoplasmic reticulum (Fig. 1B, C). The nuclei were large, paired with their flattened sides in close contact, and sometimes occupied two-thirds of the cytoplasm. The greatest sectioned nuclei measured, on average, 1.7 μm in diameter. The nucleoplasm was slightly less electron-dense than the cytoplasm. Occasionally a central nucleolus was visible (Fig. 1B). Each nucleus was bounded by an envelope consisting of two unit membranes which appeared relatively dense in the contact area (Fig. 1B).

The meronts with one diplokaryon were ovoid (Fig. 1B) or elongate (Fig. 1C). Those with two diplokarya were elongate and divided by binary fission (Fig. 1C) into two daughter cells (meronts). It is unknown if there is more than one bout of merogony. The meronts of the last generation matured into sporonts.

### Sporonts and sporogony

**Figure 2.**
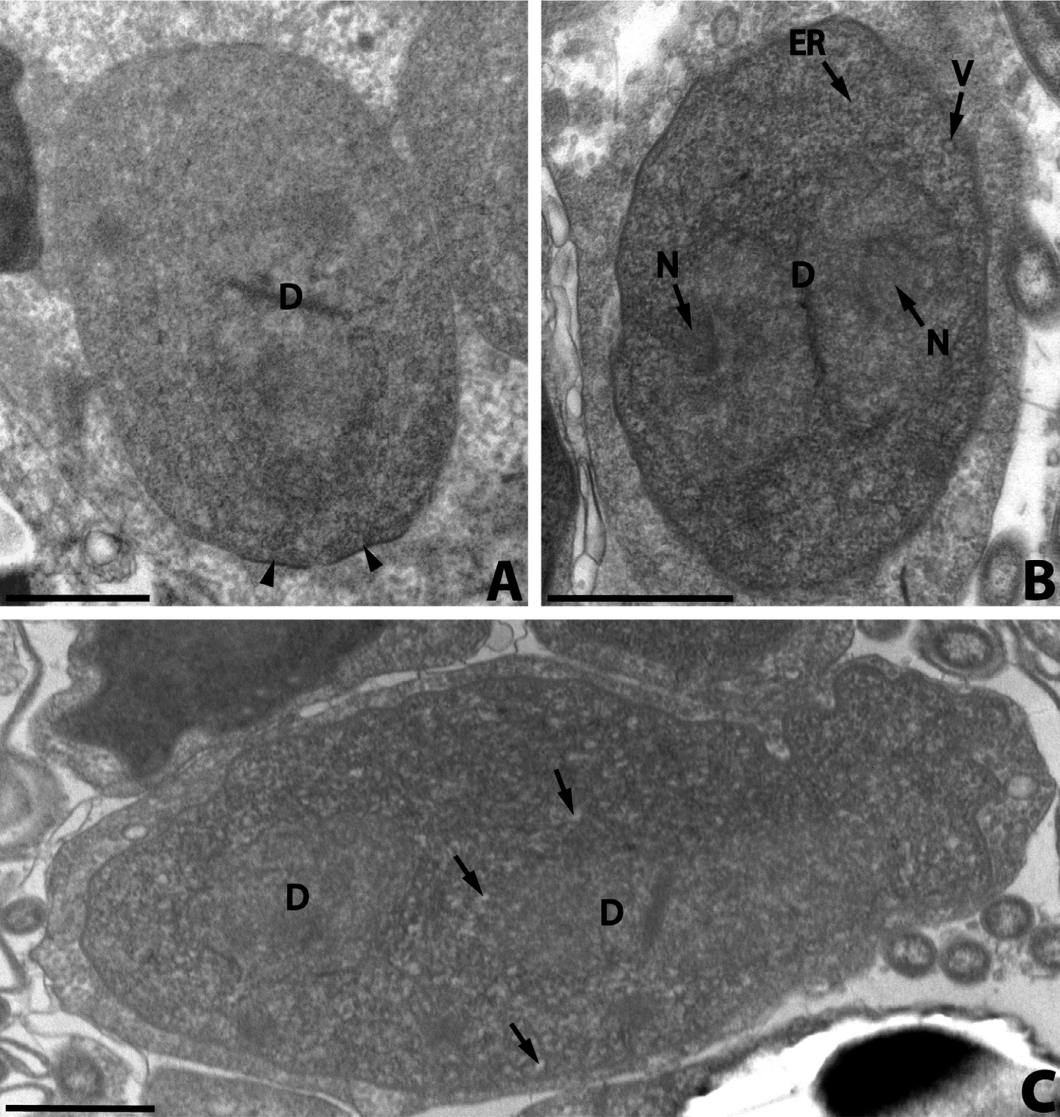
*Nosema podocotyloidis* n. sp. A. Young sporont showing the thickening of the wall (arrowheads). B. Sporont showing electron-lucent vesicles (V) and a few rough endoplasmic reticula (ER). C. Sporont with two diplokarya (D). Electron-lucent vesicles (arrows). D: diplokaryon; ER: endoplasmic reticulum; N: nucleolus-like formation; V: electron-lucent vesicles. Scale Bars: A, B, C and D, 1 μm.

At the beginning of the sporogony, the meronts of the last generation transformed with the secretion of a coat of amorphous electron-dense material on the plasmic membrane (Fig. 2A). The coat is deposited at first in irregular clumps which cover the plasmic membrane. The sporonts thus formed were bounded by a thick electron-dense wall (the thickness attained was about 20 nm), were oval (Fig. 2B) or elongate (Fig. 2C) and had one or two diplokarya. The cytoplasm of early sporonts contained numerous dispersed ribosomes, a small number of electron-lucent vesicles and a few parallel arrays of rough endoplasmic reticulum (Fig. 2B). The diplokaryon was slightly less electron-dense than the cytoplasm. It sometimes occupied more than half the cytoplasm. Nucleolus-like formations were occasionally seen (Fig. 2B).

The sporonts with two diplokarya (Fig. 2C) divided by binary fission into two sporoblasts.

### Sporoblasts and sporogenesis

**Figure 3.**
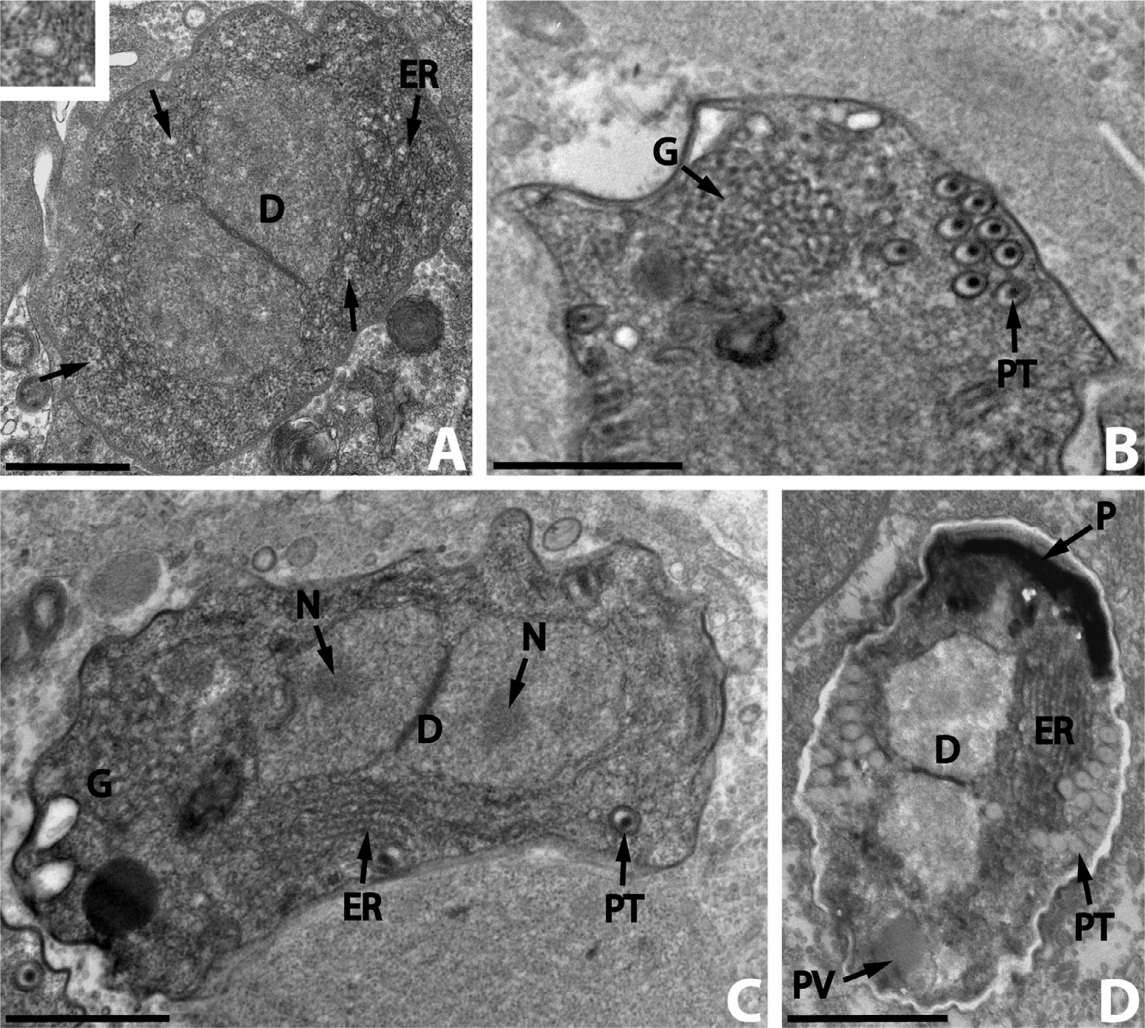
*Nosema podocotyloidis* n. sp. A. Young sporoblast showing numerous electron-lucent vesicles (arrows and insert) and the endoplasmic reticulum (ER). B. A part of the sporoblast showing the polar tube (PT) and the Golgi apparatus (G). C. Elongate sporoblast. Note the presence of the Golgi apparatus (G). D. Immature spore with 12 coils of polar tube. D: diplokaryon; ER: endoplasmic reticulum; N: nucleolus; P: polaroplast; PT: polar tube; PV: posterior vacuole. Scale Bars: A, B, C and D, 1 μm.

The young sporoblasts were ovoid cells with one central diplokaryon (Fig. 3A). They were bounded by an electron-dense coat with a thickness of approximately 30 nm. The electron-lucent vesicles of the cytoplasm increased in number and the endoplasmic reticulum became more distinct. The older sporoblasts were irregular or elongate (Fig. 3C) and their cytoplasm was full of ribosomes and lamellae of endoplasmic reticulum. The diplokaryon was less electron-dense than the cytoplasm. Distinct nucleoli were occasionally seen (Fig. 3C). The sporogenesis began with the development of a polar tube (Fig. 3B, C). Cross-sections of the immature polar tube appeared as symmetrical rings, each with an electron-dense central axis surrounded by a layer of electron-lucent material limited by a membrane (Fig. 3C). The diameter of the immature polar tube was approximately 80–120 nm. In the posterior end of some older sporoblasts, a prominent Golgi apparatus developed near the polar tube (Fig. 3B, C). Its structure was of traditional type. The polaroplast was the last spore structure formed and consisted of highly electron-dense bands (Fig. 3D). At this time, the formation of the endospore wall began. The young spores were diplokaryotic (Fig. 3D).

### Mature spores

**Figure 4.**
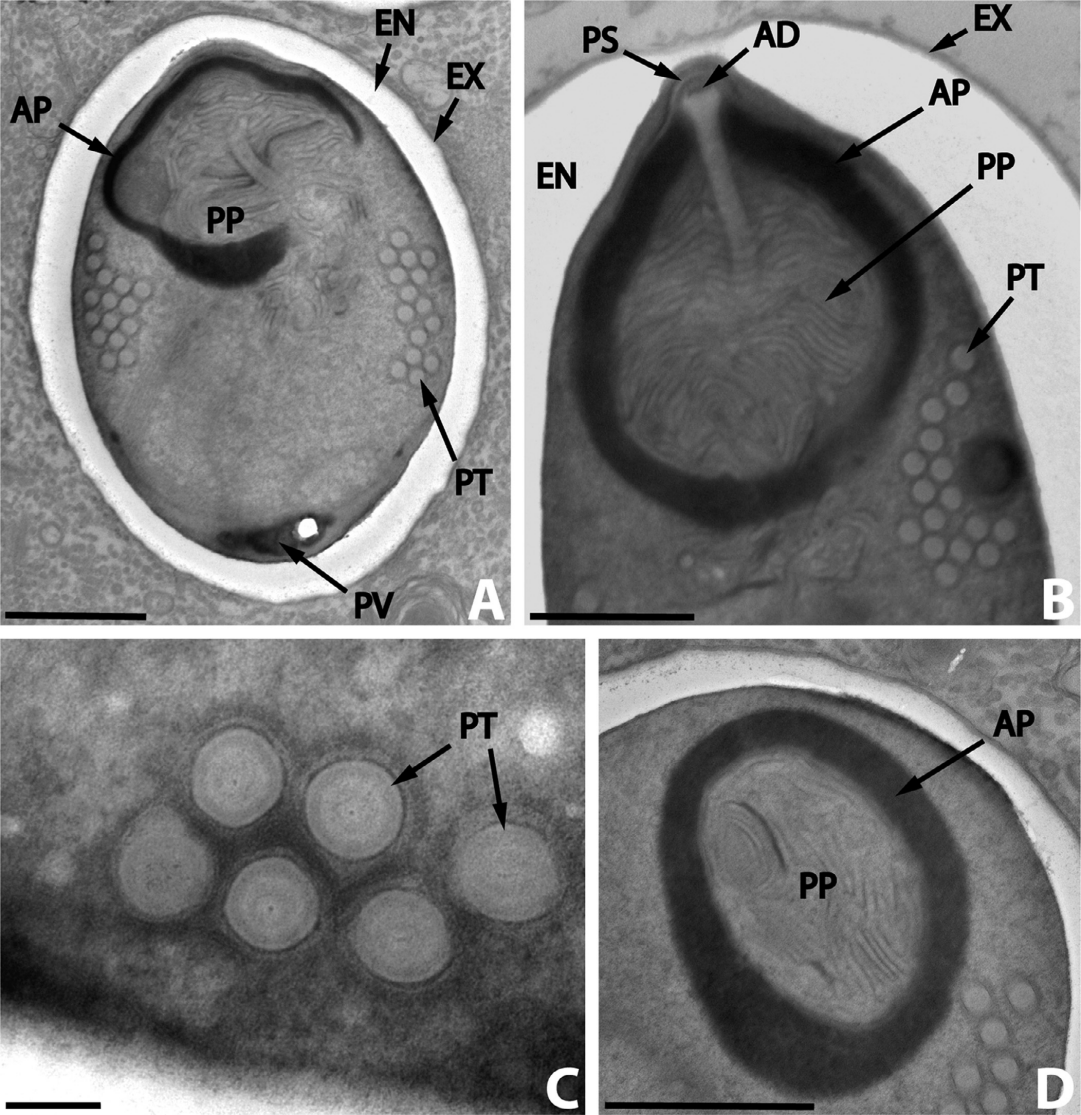
*Nosema podocotyloidis* n. sp. A. Mature spore with 15 coils of polar tube. B. Detail of the anterior region of a mature spore showing the anchoring disc (AD) and the polar sac (PS). C. Transversally sectioned polar tube coils revealing the different layers. D. Detail of the polaroplast revealing the structure of the outer or anterior region (AP) and of the inner or posterior region (PP). AP: anterior polaroplast; EN: endospore; EX: exospore; PT: polar tube; PP: posterior polaroplast; PS: polar sac; PV: posterior vacuole. Scale Bars: A, B and D, 1 μm; C, 0.1 μm.

The mature spores were broadly oval (Fig. 4A). In thin sections, the spore dimensions calculated were 3.6 (3.1–4.0) × 2.58 (1.8–3.3) μm (*n* = 25). The diplokaryon was found in the posterior part of young spores (Fig. 3D) but it was not clearly visible in mature spores.

The spore wall was about 100–300 nm thick and consisted of three parts: an electron-dense exospore, a median electron-lucent endospore and an internal unit membrane (Fig. 4A). The endospore was reduced in thickness (100–160 nm) at the anterior end of the spore (Fig. 4B).

The polar tube was isofilar with 11–16 coils, 130–155 nm in diameter, arranged in many layers in the middle region of the spore (Fig. 4A). The fine structure of the coils showed a multilayer structure (Fig. 4C). The polar tube was attached to an anchoring disc, surrounded by the polar sac (Fig. 4B). The widest sectioned disc measured 155 nm in diameter.

The polaroplast, surrounding the anterior part of the polar tube, was divided into two regions: an anterior electron-dense cup, and a posterior region, less electron-dense, composed of irregularly arranged sacs (Fig. 4A, B, D).

In the posterior part of the spore was a posterior vacuole, more electron-dense than the cytoplasm (Fig. 4A).

## Discussion

The species described here belongs to the genus *Nosema* Naegeli, 1857 as defined by Larsson [15], Sprague [22] and Canning and Vávra [9]. The following characteristics supported this generic identification:

- nuclei were paired as diplokarya at all stages of development;
- development at all stages was in direct contact with the host cell cytoplasm;
- merogony and sporogony by binary fission of diplokaryotic cells.

Eighteen species of microsporidia were described from trematodes, by Sprague [21, 22], Hussey [14], Canning [2], Canning et al. [3], Canning and Olson [8], Azevedo and Canning [1] and Levron et al. [16, 17]. Among these species, eight belong to the genus *Nosema*. They are *Nosema diphterostomi* Levron, Ternengo, Toguebaye and Marchand, 2004, *Nosema dollfusi* Sprague 1964, *Nosema eurytremae* Canning 1972, *Nosema gigantica* Canning and Madhavi 1977, *Nosema lepocreadii* Canning and Olson 1980, *Nosema monorchis* Levron, Ternengo, Toguebaye and Marchand, 2005, *Nosema strigeoideae* Hussey, 1971 and *Nosema xiphidiocercariae* Voronin, 1974 (Table 1).

**Table 1.** *Nosema* species described in the Digeneans.

*Nosema* species	Host	Hyper-host	Spore size (μm)	Number of polar tube coils	Locality	References
*N. diphterostomi*	*Diphterostomum brusinae*	*Diplodus annularis* (Fish)	2.1 × 1.4 (fixed)	6–7	Corsica France	[16]
*N. dollfusi*	Bucephalus cuculus	*Crassostrea virginica* (Mollusc)	3 × 1.7 (fixed)		Maryland USA	[21]
*N. eurytremae*	*Eurytrema pancreaticum Postharmostomum gallinum*	*Bradybaena similaris* (Mollusc)	3.94 × 2.26 (fixed)	11–12	Malaysia	[11]
*N. gigantica*	*Allocreadium fasciatusi*	*Aplocheilus melastigma* (Fish)	7.9 × 4.9 (fixed)		India	[4]
*N. lepocreadii*	*Lepocreadium manteri*	*Leuresthes tenuis* (Fish)	3.5 × 1.5 (fixed)	10	San Diego USA	[3, 7]
*N. monorchis*	*Monorchis parvus*	*Diplodus annularis* (Fish)	3.2 × 2.5 (fixed)	16–17	Corsica France	[17]
*N. podocotyloidis* n. sp	*Podocotyloides magnatestis*	*Parapristipoma octolineatum* (Fish)	3.6 × 2.58 (fixed)	11–16	Dakar Senegal	Present study
*N. strigeoideae*	*Diplostomum flexicaudum*	*Stagnicola emarginata angulata* (Mollusc)	4.7 × 3.1 (fresh)		Michigan USA	[14]
*N. xiphidiocercariae*	Plagiorchiidae	*Lymnaea palustris* (Mollusc)	4.5 × 2.3 (fresh)		Moscow Russia	[22]

*Nosema diphterostomi* was described as a hyperparasite of adults of *Diphterostomum brusinae*, an intestinal parasite of *Diplodus annularis* [16].The most distinctive characters of this species are the low number of coils of the polar tube (6–7 coils), the small diameter of the polar tube (100 nm) and the small size of the spores (2.1 × 1.4 μm).

*Nosema dollfusi* was described in the sporocysts of *Bucephalus cuculus*, a parasite of the oyster *Crassostrea virginica* from Maryland in the USA [21]. The ultrastructure of this species is unknown. This *Nosema* is differentiated from *N. podocotyloidis* n. sp. by its host which, in the larval stage, parasitises a mollusc.

*Nosema eurytremae* is a microsporidian hyperparasite of larvae of the trematodes *Eurytrema pancreaticum and Postarmostomum galilinum* in the land snail *Bradybaena similaris.* The most distinctive characters of this species are the coils of the polar tube which are arranged in a single layer close to the spore wall, the polaroplast, which consists of an anterior part composed of laminated membranes and a posterior one composed almost entirely of flattened spindle-shaped sacs, and the hosts which, in the larval stage, parasitise a land mollusc [11].

*Nosema gigantica* was found in the parenchyma of adult flukes, *Allocreadium fasciatasi*, living in the gut of the freshwater fish *Aplocheilus melastigma* from India. Its spores are ellipsoid and measure 7.9 × 4.9 μm [4]. The size of the spores of this species is different from that of *N. podocotyloidis* n. sp.

*Nosema lepocreadii* is a microsporidian hyperparasite of adult flukes, *Lepocreadium manteri*, from the gut of the California grunion, *Leuresthes tenuis*. It was studied by light and electronic microscopy [7, 8]. The most distinctive ultrastructural features of this species are:

- the diplokaryotic sporoblasts are not always produced by binary fission of the sporonts but also by multiple fission of elongate sporonts with more than two diplokarya;
- the endoplasmic reticulum is abundant in all pre-spore stages;
- the polar tube is isofilar with a maximum of 10 coils arranged in a single layer;
- the subdivision of the nuclei occurs by ingression of the inner membrane of the nuclear envelope as tongues into the nucleoplasm.

*Nosema monorchis* is a hyperparasite of *Monorchis parvus*, an intestinal parasite of *Diplodus annularis*. It exhibits the following distinctive characters: the great electron opacity and small size of the diplokarya; the polar tube is isofilar with 16–17 coils (90 nm diameter); the polaroplast presents an anterior part with closely packed lamellae and a posterior region with wider or irregularly arranged lamellae and a maximum wall thickness of 220 nm (exospore + endospore) [17].

*Nosema strigeoideae* is a hyperparasite of larval stages of *Diplostomum flexicaudum* in the snail *Stagnicola emarginata angulata* from Michigan in the USA [14]. Its ultrastructure is unknown. It is differentiated from *N. podocotyloidis* n. sp. by its spores, which measure 4.7 × 3.1 μm, and by its host which, in the larval stage, parasitises a mollusc.

*Nosema xiphidiocercariae* is a hyperparasite of sporocysts, cercariae and metacercariae in Plagiorchiidae parasites of *Lymnaea palustris*, a freshwater mollusc from Russia. The live spores measure 4.5 × 2.3 μm and the spores coloured with Giemsa measure 4.0 × 2.3 μm [22]. This *Nosema* is differentiated from *N. podocotyloidis* n. sp. by the spore size and by the fact that its host lives in a freshwater mollusc.

Therefore, we consider that *N. podocotyloidis* n. sp. is different from all other species of *Nosema* hyperparasites of trematodes.

The most prominent feature of the spores of *N. podocotyloidis* n. sp. is the anterior part of the polaroplast. It is an electron-dense cup with granular contents. This type of anterior region of the polaroplast is uncommon. It has been observed only in *Nosema lepocreadii*, a hyperparasite of *Lepocreadium manteri* [8]. Usually the polaroplast has two parts, an anterior or outer region with closely-packed lamellae and a posterior or inner region with wider and less regularly arranged lamellae or vesicular units.
